# The stigmatization dilemma in public health policy-the case of MRSA in Denmark

**DOI:** 10.1186/s12889-015-2004-y

**Published:** 2015-07-11

**Authors:** Thomas Ploug, Søren Holm, Mickey Gjerris

**Affiliations:** Centre for Applied Ethics and Philosophy of Science, Department of Communication, Aalborg University Copenhagen, A C Meyers Vænge 15, 2450 Kbh SV, Denmark; Centre for Social Ethics and Policy, School of Law, University of Manchester, Manchester, M13 9PL UK; Center for Medical Ethics, Faculty of Medicine, University of Oslo, Oslo, Norway; Section for Consumption, Bioethics and Governance, Department of Food and Resource Economics, University of Copenhagen, Rolighedsvej 25, DK-1958 Frederiksberg C, Denmark

**Keywords:** Stigmatization, Policy, MRSA, Dilemma, Ethics, Public health

## Abstract

**Background:**

Multi-resistant bacteria pose an increasing and significant public health risk. As awareness of the severity of the problem grows, it is likely that it will become the target for a range of public health interventions. Some of these can intentionally or unintentionally lead to stigmatization of groups of citizens.

**Discussion:**

The article describes the phenomenon of stigmatization within the health care area by discussing the concept in relation to AIDS and psychiatric diagnosis. It unfolds the ethical aspects of using stigmatization as a public health instrument to affect unwanted behaviours e.g. smoking. Moreover it discusses stigmatization as an unintended albeit expected side effect of public health instruments potentially used to counter the challenge of multi-resistant bacteria with particular reference to the Danish case of the growing problems with Methicillin-resistant Staphylococcus aureus (MRSA) within pig production.

**Summary:**

We argue that using stigmatization as a direct means to achieve public health outcomes is almost always ethically illegitimate. Autonomy and dignity considerations count against it, and the cost-benefit analysis that might by some be taken to outweigh these considerations will be fundamentally uncertain. We further argue that interventions where stigmatization is a side-effect need to fulfil requirements of proportionality, and that they may fall prey to ‘the stigmatization dilemma’, i.e. the dilemma that arises when all policy options are potentially stigmatizing but stigmatize different groups. When this dilemma obtains the decision-maker should choose the intervention that does not lead to permanent stigmatization and that stigmatizes as few as possible, as briefly as possible, and as little as possible.

## Background

### Introduction

Multi-resistant bacteria pose an increasing challenge to public health. In this article we consider the ethical aspects of adopting public health instruments to counter the challenge of multi-resistance that lead to stigmatization; in particular we focus on Staphylococcus Aureus (MRSA) in the Danish public health sector.

### Defining stigmatization

What is stigmatization? The locus classicus is Erving Goffman’s characterisation of stigmatization. In his interpretation, stigmatization involves the act of attributing a person with a discreditable trait that makes the person seem inferior or dangerous–ultimately something less than human–in light of an underlying ideology [1]. The ‘virtual’, social identity formed by these attributes is then subjected to various forms of discrimination resulting in the person being cut off from society [1].

Building on Goffman, a recent analysis suggests that stigmatization has five elements [2]: First, the social identification of a difference between persons and the labelling of this difference; Second, the linking of differences with negative stereotypes, i.e. linking a person to what is consciously or pre-consciously associated with undesirable characteristics; Third, the segregation of the labelled person from the labelling person, i.e. a separation of individuals into “us” and “them”; Fourth, the loss of social status and discrimination, and: Fifth, the asymmetrical distribution of power. From this perspective stigmatization therefore involves and requires power. If the labelling and stereotyping is to result in segregation and a more general loss of status, but also in more general discrimination, the labelling party must be in a position of social, economic, and political power that allows for stigmatization to happen.

All these elements can be found in historical examples of intentional stigmatization, for instance in the Nazi stigmatization of the Jewish people etc.

Note that, according to this account, stigmatization requires the presence of *all* of these elements, but also allows for each of the elements to vary in intensity, and thus for stigmatization and its effects to vary in degree. Note also that this account of stigmatization allows people to escape stigmatization in certain circumstances. If a stigmatized difference is not an essential characteristic of a person but only a contingent characteristic, then he or she may move out of the stigmatized group in so far as this characteristic changes. A stigmatized smoker may, for instance, move out of this stigmatized group and loose the stigma by giving up smoking.

Stigmatization can be both intentional and unintentional; also it may be the result of various kinds of actions, but always results in labelling, negative stereotyping, separation, loss of status and discrimination on the basis of asymmetry in power between the stigmatized and the stigmatizing party.

In the public health context the concept of stigmatization as a potential public health instrument overlaps with other concepts such as ‘denormalization’ and ‘social acceptability’ [3, 4]. From a conceptual point of view the act of ‘denormalizing’ or decreasing the ‘social acceptability’ of certain a behaviour does not necessarily lead to stigmatization of persons. However, in practice it seems likely that stigmatization–as defined above–can be the result whether this is intended or not. Neither ‘denormalizing’ nor decreasing ‘social acceptability’ necessarily involves asymmetric distributions of power but when pursued by public health authorities backed by the state, they most often do.

### Effects of stigmatization–theory and evidence

A key to considering stigmatizing instruments in the public arsenal of preventive measures is the question of the effects of stigmatization. Two questions are especially pertinent. On the one hand: “Is stigmatization effective in changing behaviour?” On the other hand: “Does stigmatization have effects that may be considered harmful to the individual?”

With regard to the first question, studies suggest that the stigmatization of smokers may indeed change smoking behavior. One study shows a correlation between what the authors call the “smoking climate”, understood as the social attitude expressed in the public sphere towards smoking, and the number of smokers [5]. More importantly, this study finds that the willingness to quit smoking is affected by experiences of “unfavourable public sentiment”. Another US study supports this conclusion by showing that the percentage of smokers in a given population depends, among other factors, on the social acceptability of smoking [6]. Both studies conclude that an increase in stigmatization or social unacceptability of smoking would be effective in decreasing smoking rates. A third cohort study of smoking cessation shows that smokers who perceive strong anti-smoking norms are more likely to try to quit smoking and are more likely to succeed in quitting [7]. On the other hand, the literature also indicates that stigmatization of obese individuals may not motivate them to change their eating or physical activity [8]. For the present purposes it is sufficient that stigmatization thus sometimes are effective in changing behaviour. If so, it is possible for public health authorities to consider employing stigmatization as public health tool in circumstances where it is likely to be effective.

In the literature there have been explicit calls for employing stigmatization as a deliberate public health instrument in relation to smoking and obesity [9, 10]. This shows that at least some authors must believe that intentional stigmatization in these contexts will be effective.

With regard to the second question, it seems obvious that it is harmful for the stigmatised, if stigmatization leads to significant loss of social status, negative discrimination etc. However, there is conflicting evidence in the literature in regard to the psychological effects on the individual. In a paper reviewing more than 20 years of research, it is concluded that prejudice against members of stigmatized groups cannot in general be demonstrated to cause lowered self-esteem among the members of these groups [11]. This conclusion is contradicted by a more recent study showing that stigmatization associated with psychiatric conditions has an influence on the self-esteem of many of the persons suffering from these diseases [12]. Several studies concerning the effects of the AIDS stigma indicate that the fear of stigmatization, in combination with experiences of being stigmatized, affect people with HIV in their choices of seeking assistance for various physical, psychological, and social needs. Another study, along the same lines, shows that the feeling of being stigmatized among individuals with HIV/AIDS is correlated with anxiety, depression, and distrust in others. Individuals who do not feel stigmatized do not suffer from anxiety, depression, or distrust in others [13]. A more recent review of the social psychology of stigma also finds that the evidence relating to the effect of stigma on self-esteem is complicated, and that the effect found varies both between studies of the same kind of stigma, between different kinds of stigmatized characteristics, and between different kinds of coping strategies [14]. The review suggests that some coping strategies that protect self-esteem may have negative effects in other important areas of life such as academic achievement and health. In conclusion, although there is somewhat conflicting evidence on the effects of stigmatization on self-esteem, there are good reasons to assume the existence of connections between stigmatization and/or perceived stigmatization and self-stigmatization and psychological harm.

## Discussion

### The ethics of stigmatization and public health intervention–three perspectives

There are many aspects of the question of ethical legitimacy concerning stigmatizing interventions to promote public health. However, three main perspectives have been developed and discussed in the literature. They relate to: 1) The utility that may be gained from such interventions [9]; 2) the violation of personal autonomy and rights [15]; and 3) the violation of human dignity that is involved in using stigmatizing interventions to promote public health [16, 17].

- Utility-perspective:
  Stigmatization of a person or a group is legitimate if and only if it is a necessary part of an intervention that maximises or Pareto-optimises the public health outcome given the costs associated with stigmatization.

The utility-perspective on stigmatization rests on a number of crucial assumptions that must be analysed from case to case. *Firstly*, that the benefits and costs of stigmatization can be known and compared. Given the range of negative effects of stigmatization, the possibility of individual differences in the response to stigmatization, and not least that the means of stigmatization, may vary in the severity and duration in which they expose an individual to stigmatization, this seems a particularly questionable assumption. *Secondly*, that the benefits and costs of the intervention can be estimated when the issue of stigmatization is bracketed. *Thirdly*, that there are no alternative and less stigmatizing ways of obtaining the same benefits in public health. Note, *finally*, that the utility-perspective may be given two interpretations depending on whether maximization or optimization of public health is chosen as the outcome criterion. Maximization requires that the overall gain in public health is greater than for any alternative intervention. Optimization requires that the overall gain in public health is greater than for any alternative intervention and that no person is made worse off. Optimization is the stricter criterion in relation to stigmatization, because it implies that a stigmatized person also should obtain significant gains in health in order to counterbalance the costs of suffering stigmatization.

- Autonomy-perspective:
  Using stigmatizing interventions for public health purposes rests on state-sanctioned paternalism, i.e. the view that it is legitimate for the state to influence the choices of individual citizens with the aim of benefitting them. Protection of personal autonomy implies that state-sanctioned paternalism is illegitimate. More specifically, we shall say that the autonomy-perspective implies that the individual’s right to pursue his or her own goals without undue influence must take priority in issues regarding public health policies to the widest possible extent, and in particular where the public health intervention will subject an individual to harm or risk of harm.

It has been argued that the use of stigmatizing interventions to promote public health is in line with existing forms of state-sanctioned paternalism used in the public health sector such as prohibitions against injecting very dangerous drugs etc. [18]. However, although prohibition clearly is a form of state-sanctioned paternalism, there is a crucial ethical difference between such examples and the use of stigmatizing interventions. Stigmatization is–contrary to prohibition–known to cause significant risk of harm to the individual in the form of discrimination, negative psychological effects, and may even affect physical wellbeing by affecting the motivation to seek medical assistance. From a perspective focusing on the autonomy of the citizen, it seems a plausible position that state-sanctioned, paternalistic, public health interventions are justified only to the extent that they do not cause harm to the individual. Any decision to subject an individual to interventions aimed at improving health but known to cause harm or risk of harm, must be taken by the individual.

- Dignity-perspective:
  Stigmatization operates by dehumanizing individuals. Treating others as human beings is an expression of a basic respect for individuals [16]. Stigmatization is therefore ethically illegitimate, and renders interventions that create stigmatization ethically illegitimate.

The dignity perspective points to the degrading and humiliating character of stigmatization. Stigmatization operates in part by pointing to the undesirability of certain aspects of the other person-aspects that are seen as deviant and/or shameful. Going back to Goffman, it is not only certain actions, but even the person herself that is being found less worthy than those not being associated with the perceived negative aspects. Such stigmatization violates the fundamental value that all people are of equal and infinite worth. In this perspective the alleged fact that stigmatization may be a recurring phenomenon in society cannot justify the proactive use of stigmatization by the state.

The three basic perspectives are in tension with one another, and a choice to use stigmatizing measures to promote public health must balance these values and address underlying assumptions. The principle of proportionality and the precautionary principle are of relevance for this process. The principle of proportionality requires that there is a reasonable proportionality between the measures used and their outcome [19]. In our case this amounts to a requirement of reasonable proportionality between the people affected by stigmatization and the health benefits achieved by the stigmatizing intervention. Let us first consider interventions that primarily work through stigmatization. Given the uncertainty about the effectiveness of stigmatization as an instrument of public health, but also the character, extent, and likelihood of negative effects on individuals, any attempt to secure proportionality between the intervention and its effects are questionable, because of the problems in estimating benefits and costs. This clearly lays the ground for the precautionary principle. Although strongly contested with regard to its validity as a decision-making principle for public policy decision, the precautionary principle requires that precaution is exercised where there is uncertainty about the possibility, size, and probability of potential negative effects of a given intervention [20, 21]. The precautionary principle seems pertinent for any consideration of using stigmatization, since the use of stigmatization is associated with harmful effects, but there is uncertainty about the size of these.

We believe that the uncertainty of the effectiveness and of the costs to the individual of using stigmatization to promote public health, in combination with the considerations related to state-sanctioned paternalism and the challenges to human dignity, represent a strong case for considering stigmatization as an ethically illegitimate instrument of public health interventions.

Often, however, stigmatization is not the primary means by which a public health intervention works, but rather a desired or undesired side effect. Here the analysis becomes more complicated, since there will be cases where reliable empirical knowledge points to very significant benefits of the intervention that are likely to outweigh any harm caused by the stigmatization. Such an intervention would thus fulfil the maximisation criterion.

In the following we would like to deepen the analysis by considering a particular case where the public health authorities are faced with choosing between different public health policies to combat a threat to public health in a context where one or more groups of people will be stigmatized given the prevalent public attitudes towards the problem in question.

### The case of MRSA in Denmark

#### Multiresistant bacteria

Multi-resistant bacteria are a growing problem around the world. The World Health Organization defines antimicrobial resistance as “*resistance of a microorganism to an antimicrobial drug that was originally effective for treatment of infections caused by it*” [22]. The seriousness of the issue has been placed in perspective by the British Government’s chief medical officer, Professor Dame Sally Davies, who talks of the growing resistance to antibiotics as a ticking time bomb. She furthermore states that the problem should be “*ranked along with terrorism on a list of threats to the nation*” [23].

The development of resistance to antibiotics in bacteria is a naturally occurring evolutionary mechanism, which benefits the different bacteria such that the individual strains become immune to the antibiotics used against them. However, according to WHO, this development is accelerating, thus rendering existing antibiotics useless against the new strains of bacteria that are developing in response to widespread and increased human use of antibiotics [22].

Antibiotics are used against a wide variety of microbial infections in humans ranging from life-threatening situations in relation to e.g. prematurely born children, surgery, and cancer, to less serious problems such as e.g. impetigo, acne, and tonsillitis. Furthermore antibiotics are used for animals for a variety of reasons. The use for companion and production animals is the most widespread. The reasons behind the spread of multi-resistant bacteria and the best avenues to solve or at least mitigate the problem are therefore complex [24, 25].

#### MRSA in Denmark

Our case is from Denmark. It pertains to the combination of multi-resistant MRSA CC398 bacteria (known as “Swine-MRSA” in the Danish public) from the use of antibiotics in the Danish pig production and public health system. In 2013 in Denmark almost 80 % of all antibiotics used on animals were used in pig production [26]. The number of reported cases of MRSA CC398 in humans has likewise grown from 12 in 2007 to more than 1.000 in 2014 [27]. Since 2012, 5 deaths have been reported as being directly connected to the bacteria [28].

In 2012 two journalists–by referring to the Danish act on public access to official records-applied to see which pig farms had been tested positive with regard to MRSA CC398. The Danish Veterinary and Food Administration rejected this request, as it would–among other things–allegedly place the affected farmers at risk of being socially stigmatized. The case is complicated from a legal point of view, since the two journalists simultaniously acquired the information through an anonymous source, and subsequently published the data. This resulted in them being convicted with reference to the law protecting personal data. Later the Danish ‘Ombudsmand’ ruled that the interest of the public outweighed the risk for the affected farmers and the Danish Veterinary and Food Administration were told to grant access to the information. This decision has then been appealed by the Danish Agriculture and Food Council that represents the producers [29]. As can be readily seen the legal side of the case is more than complicated. Our focus is, however, on an ethical analysis of the values embedded in the question of stigmatization in relation to MRSA CC398, and whether *not* making the information publically available does not also carry the risk of stigmatizing certain citizens.

### The ethics of MRSA interventions–the choice of whom to stigmatize

#### The scenarios

As mentioned above, one of the reasons for not publishing information on which farms are infected by MRSA CC398 has been the fear of stigmatizing the farmer and people involved in the production (family, employees etc.) to avoid risks of harming them socially and economically.

Based on the effects of providing the public with knowledge about the infected farms, four scenarios may be distinguished. The first is where public knowledge about which farms are infected actually increases public health by reducing the number of persons who get infected [1]. The second is where the knowledge does not change public health [2]. Since people related to farms allegedly may come to experience stigmatization as a result of the public knowledge of the MRSA CC398, scenario 1 and 2 may be further divided based on the occurrence of stigmatization (S) or no stigmatization (N). We thus end up with the following matrix (Table 1):

**Table 1:** Public Health Benefits and Stigmatization

Knowledge->	Stigmatization	No Stigmatization
Public Health Benefits	1S	1N
No Public Health Benefits	2S	2N

From a utility perspective the key scenario is 1S where the public health benefits must be weighed against the harm suffered by the stigmatized farmers. This scenario is evidently controversial as seen from an autonomy and dignity perspective. Also it may be controversial from a utility perspective, depending on the size of the public health gain. On the other hand, scenario 1N is uncontroversial from all perspectives since there are public health benefits to be gained and no stigmatization. Both scenarios 2S and 2N hold no gain in public health and therefore cannot be justified within a utility perspective narrowly focused on public health. Scenario 2S–contrary to 2N–involves stigmatization and therefore runs counter to protection of autonomy and dignity.

In light of this preliminary analysis, we shall focus on the justifiability of scenario 1S. Note firstly that it cannot be justified within a utility perspective that trades on optimization, since the farmers that suffer stigmatization do not gain the direct health benefits. We here assume that they already know about the infection and therefore must be assumed to exhibit behavior in terms of their health that is not likely to change because of the dissemination of the relevant information. They are thus made worse off by the stigmatization ensuing from the dissemination of the relevant information. There may, hypothetically, be other long term benefits for the farmers if disclosure leads to increased health care support for them personally, but such benefits are uncertain and it is an open question whether or not they would outweigh the costs associated with having been stigmatized. Thus the real question concerns whether scenario 1S may be justified from a utility maximizing perspective. In order to decide this we must address the main assumptions of this scenario, which are that the act of making information on infected farms publicly available will ensue in stigmatization of the farmers, but also that it will be associated with improvements to public health. There is some evidence in favor of both of these assumptions.

The potential effects of being stigmatized in general have been described above in relation to HIV/AIDS and psychiatric disorders. Similar effects must be expected in people infected with MRSA CC398. A Swedish study from 2009, based on qualitative interviews with 13 MRSA (not MRSA CC398) patients, uncovered that the patients shared experiences of feeling “invaded, insecure, and alone” because of the infection. Common findings were guilt and shame in relation to close relatives. Some spoke of social withdrawal, work-related problems, fear and experiences of social isolation etc. [30]. These findings are compatible with stigmatization having taken place, but since there are also other possible explanations they are not absolutely conclusive. However, stories and interviews in the Danish media with MRSA CC398 infected persons seem to indicate that stigmatization is taking place. One former production leader from a pig production facility reports that his experiences with the health care system in relation to a knee operation and the birth of his child made him feel like a leper, because of the different precautions that had to be taken to avoid him or his girlfriend passing on the infection [31]. There are also reports of families feeling stigmatized and socially excluded from local communities, even refusals to shake hands. Stories like these are not necessarily representative, but there is no doubt that they reinforce the experience of stigmatization in others who are infected. There is thus little doubt that being infected with MRSA CC398 can lead to an experience of stigmatization. In the Danish media there has also been discussions about the ethical/causal responsibility of the farming sector as a whole for producing multi-resistance. In these discussions the responsibility has primarily been seen as sector-wide, and not individualised in relation to the farmers. It is worth noting, that being infected with MRSA CC398 does not necessarily lead to stigmatization. One can imagine a situation where the Danish public is supportive of the infected people instead. This is, however, not the picture that can be drawn from the media coverage in Denmark of the issue.

The gain in public health following public availability of information on infected farms may be achieved in at least two ways. Firstly, it may follow from increased efforts from farmers to combat the infection in order to avoid stigmatization. Secondly, it may follow from the ability to avoid sources of infection through the awareness of these.

In terms of the former, we have previously reported the results of two studies indicating that stigmatization of smokers or increase in the social unacceptability of smoking would be effective in decreasing smoking rates [5, 6]. Although there are significant differences between combatting smoking and MRSA infected farms, one could argue that the stigmatization of farmers basically creates an incentive for changing a state of affairs that may be changed by the efforts of the farmer. MRSA–like smoking–is to some extent under the influence and control of the farmer.

In terms of health benefits related to a general awareness of specific infection sources, the case is slightly more complex. According to Danish health authorities the risk of being infected with MRSA CC398 when working in an infected pig production facility is significant. There is also a significant, albeit smaller risk of passing the infection on to close contacts such as partners and children. The risk of catching the infection by an everyday encounter with an infected person or by visiting a pig production facility is, however, considered to be insignificant [32, 33]. That it is possible to catch MRSA 398 without working in the pig industry is, however, evident, as at least 4 of the 5 deaths ascribed so far to MRSA CC398 in Denmark involve people external to a pig production facility [34]. The general conclusion seems to be that if workers are not informed that they work at an MRSA facility, there is a significant risk that they will carry it home to their relatives that again might carry it on in their line of work. Thus, the health benefit is linked to awareness–not of the public in general–but of the farmer and employees at farms. The general public will only gain few health benefits from knowing that the pigs of pig producer A have MRSA.

Although there is some evidence of both the stigmatizing effect and some public health benefits following the public availability of information on MRSA infected farms, we believe the evidence to be insufficient for making any definite conclusions. In any case, if public knowledge about infected farms should result in stigmatization, we shall, in accordance with our previous analysis of the general issue of using or allowing stigmatization for public health purposes, defend the stance based on the autonomy and dignity perspective, i.e. that stigmatization cannot be considered a legitimate instrument to promote public health. This conclusion does not, however, take into account a very important, but easily overlooked, feature of the discussion of using or allowing stigmatization for public health purposes.

#### The stigmatization dilemma

So far we have considered the ethics of using measures to prevent the spread of disease that may result in stigmatization of a group of people. Having acknowledged the ethical relevance of stigmatization, the argument is, however, still too simple. Often the choice to be made in the public health context is not between stigmatization and no stigmatization, but rather between who or which group of people to stigmatize. That is, public health intervention is in effect faced with a choice between an intervention that will stigmatize one group and an intervention or a non-intervention that will stigmatize another group or several other groups. If this is the case, it is not clear, as we will argue, who has the right not to or deserves the most not to be stigmatized. The MRSA case given above illustrates this well.

In the current situation, some people are already infected with MRSA CC398 and may as a consequence suffer stigmatization when identified; those patients, irrespective of whether they are somehow related to the pig-industry or not, are treated in a special way in the Danish health sector to avoid spreading the infection. It is clearly stated in the guidelines from the Danish health authorities that all citizens-irrespective of their MRSA status-are entitled to the same care from the public health sector [32]. They are, however, encouraged-as long as they are considered carriers of the bacteria-to inform all health personnel they come into contact with that they have been tested positive and are issued with a small ID-card containing this information. Besides being asked to do this the health authorities recommend that everybody in the household are treated as carriers of MRSA, undergo an eradication treatment, and are tested for MRSA afterwards. Furthermore the patient is instructed to thoroughly clean his or her home, being given specific guidelines for how to go about this.

Stigmatization following the diagnosis of MRSA is related to several of the directives from the Danish health authorities. First, there is the identification of a difference between carriers and non-carriers and the labeling that is related to the diagnosis in itself, but also-and not least-the requirement of carrying identification as being MRSA infected. This implies that the patient is seen as a threat to public health and may readily lead to the patient seeing him or herself as such. Secondly, providing the information that MRSA patients carry equal rights to treatment, but in the same breath encouraging them to identify themselves as carriers of MRSA, would seem to have two implications; it may enhance the experience of stigmatization although it is aimed at reassuring the patients, but it can also be read as suggesting a conditional right to treatment based on the extent to which one poses a threat to the health of others or conditional on the extent to which one identifies oneself as a carrier of MRSA in interactions with the health care system. Both of these interpretations may result in a stronger experience of stigmatization. The carriers of MRSA are thus treated differently than most other patients in the healthcare system, e.g. by being required to carry ID. Thus, if the information provided to the patients and the recommendations issued subsequently have the implications discussed above, it seems reasonable for the patient to fear discrimination. Thirdly, the fact that the whole of the family is to undergo an eradication treatment and their house is to be cleaned in a specific way widens the social implications of contracting MRSA. The threat from MRSA does not only come from a particular person, but the family and household of that person. It seems that this is likely to increase the social costs of carrying MRSA, not least for children and youths–at least for a period of time. Their social status can be affected negatively to the extent this becomes known or to the extent the requirement to keep it a secret, affects the children. Thus, stigmatization and self-stigmatization are both possibilities here.

It should be noted that all of these discriminatory effects must be expected to increase with a growing number of people contracting MRSA. As mentioned above: more than 1.000 people have been diagnosed as carriers of MRSA CC398 in 2014 in Denmark. It is also worth noting that there is evidence that an MRSA diagnosis can cause psychological and social problems for those who experience it [35]. Also, the harm to the individual caused by stigmatization can be a result of actual stigmatization in the public sphere or related to the individual perceiving stigmatization, whether this actually takes place or not. What we are arguing here is that the precautionary measures dictated by the Danish health authorities are likely to accommodate this latter kind of stigmatization where the individual is not stigmatized “officially”, but nonetheless experiences being stigmatized. As already mentioned, it is very clear in the guidelines from the authorities that there is to be no difference in the care offered to carriers and non-carriers. Note finally, that we do not–so far–suggest that the Danish authorities’ precautionary measures are in any respect wrong, but only that may lead to stigmatization/the feeling of being stigmatized among the group of people carrying MRSA.

The pig producers in general may also experience stigmatization. Thus, not making public which farms are infected and which are not, carries the risk of stigmatizing even the pig producers who do not have MRSA CC398. This illustrates that merely identifying a public health problem, e.g. “there is a MRSA problem in Danish pig production”, may have some stigmatizing effect. The level of this concern is obviously related to the number of infected/non-infected farms, but as mentioned above there is a growing numbers of reports showing that many pig producers experience social stigmatization even though it is not clear whether they are infected or not [31, 36].

If public health interventions may–as substantiated in this section–have unintentionally stigmatizing effects on different groups, the question of the legitimacy of using such measures in public health interventions becomes more complex. A particularly complex situation arises if it is possible to avoid the stigmatization of one group–or partly avoid or diminish it–by using measures that will inevitably lead to the stigmatization of another group. In that case we are faced with a stigmatization dilemma. The MRSA case could be argued to represent such a dilemma. As we have seen there is some ground for believing that by making information on MRSA infected farms publicly available there are potential gains in public health, but at the cost of the farmers suffering stigmatization. Failure to make the information publicly available may result in more citizens contracting MRSA and consequently suffering stigmatization. Add to this the stigmatization that may be suffered by farmers on non-infected farms, and it is clear that public health interventions in this field are likely to be faced with a stigmatization dilemma. We have previously argued and maintained that stigmatization must be avoided, if at all possible. However, when facing a stigmatization dilemma the question is no longer if it is legitimate for public health interventions to employ stigmatizing measures, but rather whom public health interventions would be most justified in stigmatizing.

In clonclusion it seems that stigmatization dilemmas are likely to occur in relation to surveillance and prevention of infectious diseases because it is difficult to communicate effectively about infectious threats to the public health without running the risk of associating those who are infected with negative stereotypes.

#### The ethics of “choosing” whom to stigmatize

The stigmatization dilemma arising in the case of MRSA in Denmark reinvigorates the debate on the ethical difference between acts and omissions. One way to address the issue would thus be to let this distinction be decisive for the choice between the horns of the dilemma. A defender of this distinction would claim that it is worse to intentionally act in ways that bring about a certain negative outcome than to intentionally omit actions with the same outcome. In the MRSA case the Danish health authorities may intervene in ways that directly result in the stigmatization of farmers and others, e.g. by making information on infected farms publicly available, but the failure to intervene may also result in stigmatization, e.g. of the infected citizens. In light of the MRSA case this could be taken to imply that it must be considered a worse act by the health authorities to impose stigmatization on the farmers as the aim of a public health intervention than to omit such actions with the stigmatization of citizens as a result. In both situations stigmatization of a group of people is the outcome, but in the former case it is the outcome of an act, in the latter case it is the outcome of an omission. Note that the negative outcome of act and omission is the same in light of an underlying value-theory that defines stigmatization as wrong per se. The defender of this view would thus have to base it on considerations that entail the wrongfulness of stigmatization as such, e.g. considerations from within the autonomy or dignity perspective, and would also have to reject considerations from within the utility perspective since they may ultimately lead to the rejection of the distinction between acts and omissions.

Alternatively one could argue that the distinction between acts and omissions cannot be upheld–at least not in certain cases. This view would correspond with an underlying value theory that credits considerations from within the utility, autonomy, as well as the dignity perspective in that it upholds the unacceptability of stigmatization as such, but allows for considerations of utility to override autonomy and dignity considerations in certain cases. The cardinal question then becomes what features a situation should exhibit in order to make a choice of intervention involving stigmatization justified? A situation characterized by a stigmatization dilemma could be claimed to be such a case.

Thus, the proviso on any form of active use of health interventions knowingly resulting in the stigmatization of groups of people would be that it is the outcome of a choice between different courses of actions and omissions that all imply the stigmatization of a group of people. It must be a choice between whom to stigmatize. Note that this covers situations in which the stigmatization on one horn of the dilemma is not directly or indirectly related to the public health intervention, but may result from failing to act. Thus, it includes situations where the failure to make a public health intervention with stigmatizing effects will result in foreseeable informal, non-institutionalized stigmatization of a group of people. A failure to make a public health intervention with stigmatizing effects directed at people with e.g. leprosy could result in increased socially driven stigmatization. Second, it seems evident that stigmatization can only be legitimately used where it is likely to have an effect on public health. Any attempt of improving public health by means involving the stigmatization of people must happen on the basis of evidence for the positive effect on public health of the interventions involving stigmatization [9]. Thirdly, it must be possible to escape stigmatization. That is, it must be possible for the group of people suffering stigmatization to change the aspect of their situation that triggers the stigmatization. In other words, it must be possible to obtain the public health benefit without permanently placing people in a stigmatized position. Relatedly, any intervention resulting in stigmatization should employ means that stigmatizes as few as possible, as briefly as possible, and as little as possible.

A special case may obtain when the public health intervention is faced with a stigmatization dilemma, where one of the groups threatened by stigmatization by health authorities is stigmatized already. This may in itself provide a reason not further to stigmatize this group [37].

## Summary

It is uncontroversial to claim that some public health interventions cause particular groups and individuals to become stigmatized, and that this stigmatization may be harmful psychologically, or because it leads to harmful discrimination. The stigmatization may either be a deliberately chosen means to achieve a positive public health outcome, or it may be an intended or unintended side effect of a public health intervention where the positive outcome is not solely dependent on the stigmatizing effect.

In this paper we have argued that using stigmatization as a direct means to achieve public health outcomes is almost always ethically illegitimate. Autonomy and dignity considerations count against it, and the cost-benefit analysis that might by some be taken to outweigh these considerations will be fundamentally uncertain.

We have further argued that interventions where stigmatization is a side-effect need to fulfil requirements of proportionality, and that they may fall prey to ‘the stigmatization dilemma’, i.e. the dilemma that arises when all policy options are potentially stigmatizing but to different groups. When such a dilemma arises the decision-maker should choose the intervention that does not lead to permanent stigmatization and that stigmatizes as few as possible, as briefly as possible, and as little as possible.
